# Potential Geographical Distribution Prediction and Ecological Niche Change Analysis of *Pissodes castaneus*

**DOI:** 10.3390/insects17101002

**Published:** 2026-09-28

**Authors:** Yuxuan Li, Dongrui Sun, Yiwen Wang, Zhuojin Wu, Te Liang, Juan Shi

**Affiliations:** 1 Beijing Key Laboratory for Forest Pest Control, Beijing Forestry University, Beijing 100083, China; 2 Sino-France Joint Laboratory for Invasive Forest Pests in Eurasia, Beijing Forestry University, Beijing 100083, China; 3 Inner Mongolia Forestry Research Institute, Hohhot 010011, China

**Keywords:** *Pissodes castaneus*, MaxEnt, climate change, potential distribution, niche dynamics analysis

## Abstract

*Pissodes castaneus* is an internationally quarantined pest that damages various pine species. The global distribution patterns and invasion potential of *P. castaneus* have not been systematically assessed, thus leaving relevant prevention and control systems without a theoretical foundation. In this study, we used an optimized MaxEnt model to predict the global suitable areas of *P. castaneus* under current and future climate conditions. At the same time, it assesses the changes in the ecological niche of *P. castaneus* under climate change. The results indicate that the AUC values of the MaxEnt model exceeded 0.9 under both current and future climate scenarios, indicating high predictive accuracy. Under current climate conditions, the total global potential suitable area for *P. castaneus* is 1.08 × 10^7^ km^2^. The highly suitable areas are mainly distributed in Western Europe, northern Turkey, southeastern Australia, the west coast of North America, and the east coast of South America. Under the two future climate scenarios (SSP126 and SSP585), the total suitable area initially decreases and then increases, reaching its maximum during 2081–2100. Compared with current conditions, suitable areas shift northward in the Northern Hemisphere. Under both the SSP126 and SSP585 scenarios, although its niche remains highly conservative relative to current conditions, different emission scenarios may affect the niche dynamics of *P. castaneus.* This study reveals the spatiotemporal dynamics of suitable areas and niche characteristics of *P. castaneus* on a global scale, providing a basis for worldwide quarantine and inspection efforts and offering scientific data support for future prevention and control of its spread.

## Simple Summary

*Pissodes castaneus* is an internationally quarantined pest that damages various pine species. In this study, we used an optimized MaxEnt model to predict the global suitable areas of *P. castaneus* under current and future climate conditions. At the same time, it assesses the changes in the ecological niche of *P. castaneus* under climate change. Under current climate conditions, the highly suitable areas of *P. castaneus* are mainly distributed in Western Europe, northern Turkey, southeastern Australia, the west coast of North America, and the east coast of South America. Under the two future climate scenarios, the total suitable area initially decreases and then increases. Under both the SSP126 and SSP585 scenarios, although its niche remains highly conservative relative to current conditions, different emission scenarios may affect the niche dynamics of *P. castaneus*. This study reveals the spatiotemporal dynamics of suitable areas and niche characteristics of *P. castaneus* on a global scale, providing a basis for worldwide quarantine and inspection efforts and offering scientific data support for future prevention and control of its spread.

## 1. Introduction

As one of the most widely distributed and economically valuable coniferous genera globally, *Pinus* spp. plays an irreplaceable role in supporting the forestry industry and maintaining ecosystem functions [1]. The global forest area amounts to 4.14 billion hectares [2] with coniferous trees representing the most widely planted group and accounting for approximately 39% of the world’s forests [3,4]. *Pinus* species are planted in dozens of countries, including China, the United States, Brazil, and New Zealand, forming the mainstay of industrial plantations in temperate and subtropical regions and serving as a major resource for construction, papermaking, furniture manufacturing, and bioenergy fields [5,6,7]. Additionally, *Pinus* trees defend against pests and diseases through a complex resin defense system, which not only supports forest health but also underpins the global rosin and turpentine industry [8,9]. Furthermore, pine forests also make significant contributions to maintaining ecosystem functions. According to global surveys, *Pinus* accounts for a total volume of 5.4046 × 10^10^ m^3^, representing 8.6% of the world’s forest volume making it the largest genus by forest volume globally [2], and constitutes a significant carbon reservoir.

*Pissodes castaneus* (De Geer, 1775), belonging to the order Coleoptera, family Curculionidae, and genus *Pissodes*, primarily attacks *Pinus* species, including *Pinus taeda* L. and *Pinus sylvestris* L. etc, and can also damage other conifers such as *Picea abies* and *Abies alba* [10]. Adults of *P. castaneus* feed on the bark of many coniferous trees, which leads to the weakening and eventual death of young trees; the larvae feed beneath the bark and also on terminal branches and apical buds; Feeding beneath the bark often leads to rapid tree mortality within weeks—resulting in substantial economic losses [11]. In addition, *P. castaneus* can carry root-rotting fungi, such as the rust fungus and *Cronartium pini* (Willd.) Jørst., causing root rot and ultimately tree wilting and death [12,13,14,15].

*P. castaneus* is native to Europe and has successfully invaded South American countries including Argentina [16,17], Brazil, Uruguay and Chile [18]. In many European countries (e.g., France, the United Kingdom, Russia, Finland, and Spain), *P. castaneus* is an important pine pest, causing significant damage to young trees aged 2–15 years and leading to high mortality in young plantations. In Portugal, infestation by *P. castaneus* in seed production plantations can severely reduce yield [19], and it has also caused serious losses in natural and planted forests across southeastern Europe and the eastern Mediterranean [20]. In Brazil, *P. castaneus* is currently a major pest of *Pinus* (Pinaceae), since its appearance as an invasive species in 2001, it has caused severe damage [21]. For instance, in 2002, 7.6% of the *P. taeda* in Cambará do Sul, Rio Grande do Sul state, were affected, and in the vicinity of Santos Jacó, Santa Catarina state, 16.5% were also impacted [18]. *P. castaneus* is characterized by a wide host range, a concealed life cycle, and the ability to spread over long distances with logs and unpeeled wood. The European and Mediterranean Plant Protection Organization (EPPO) has assessed it as posing a high risk of introduction and recommends regulating seedlings and planting materials. In recent years, this pest has been intercepted multiple times in non-native regions such as Asia and South America, posing a serious biosecurity threat to global wood trade [22,23]. The Southern Cone Plant Health Committee, as well as countries such as Ecuador, the United States, Turkey, and China, have classified it as a quarantine pest.

Species Distribution Models (SDMs) have become important tools for predicting species’ geographical distribution and their responses to climate change. Among them, the MaxEnt model based on the principle of maximum entropy, is widely used in the risk assessment of invasive species, early warning of pest spread, and biodiversity conservation planning due to its requirement for only species distribution point data, robustness to small sample sizes, and excellent predictive performance [24,25,26]. By integrating species occurrence records with environmental variables, MaxEnt model can effectively simulate potential changes in species distribution. Since its inception, the model has been continuously updated, with innovations including its combination with deep learning [27] or the incorporation of phylogenetic information [28]. In addition, the model has seen algorithmic and operational refinements, while its application in climate change research has expanded. It has become one of the most reliable and efficient tools for predicting suitable habitats for species.

To date, no systematic study or assessment has been conducted on the global suitable habitat distribution pattern and invasion potential of *P. castaneus*, and the effects of climate and environmental change on its potential geographic distribution and ecological niche remain unclear. Consequently, the relevant control system lacks a theoretical basis. In addition, the key assumption of niche model is that species are niche conservative and occupy similar environmental conditions in a new geographical range or time period [29]. In this study, we hypothesized that *P. castaneus* is niche conservative under climate change and used MaxEnt model to predict its potential geographical distribution under current and future conditions. Then we also identify the key environmental variables affecting its distribution. At the same time, it assesses the changes in the ecological niche of *P. castaneus* under climate change. The goal is to provide key data support for establishing a cross-border biosecurity early warning system and formulating differentiated control strategies for this pest.

## 2. Materials and Methods

### 2.1. Collection of Distribution Data and Climate Variables

In this study, a total of 2880 distribution data of *P. castaneus* were collected from the Global Biodiversity Information Facility (GBIF Occurrence Download. Available online: https://www.gbif.org/, accessed on 9 July 2025) and iNaturalist (https://www.inaturalist.org, accessed on 9 July 2025). The “location blurring” function in iNaturalist substitutes actual coordinates with randomly generated points inside 0.2° × 0.2° cells, which may create spatial bias in species distribution records. In addition, when redistributing such blurred records, other open databases (e.g., GBIF) leave them uncorrected, causing mistakes in compiling geographic distribution information [30]. To limit this problem’s influence on our analysis as far as possible, iNaturalist records were manually checked, and points with blurred coordinates were removed. The quality grade of iNaturalist records was set to “research grade” to improve data accuracy.

Global environmental layers came from WorldClim (www.worldclim.org), comprising 19 bioclimatic variables (bio1–bio19; Table S1) for 1970–2000. Future climate layers were also sourced from WorldClim. To model the potential future distribution of *P. castaneus*, we used the Beijing Climate Center Climate System Model (BCC-CSM2-MR) under the Coupled Model Intercomparison Project Phase 6 (CMIP6) to produce projections for four intervals (2021–2040, 2041–2060, 2061–2080, and 2081–2100) and two shared socioeconomic pathways (SSPs): SSP126 (low forcing) and SSP585 (high forcing) [31]. Compared with the versions used in CMIP5, the BCC-CSM2-MR in CMIP6 has significantly enhanced its ability to simulate key climate variables such as global mean surface air temperature, sea surface temperature, atmospheric circulation, and sea ice extent and thickness [32].

### 2.2. Data Processing

#### 2.2.1. Distribution Point Screening

Climatic variables used here were at 2.5 arc-minute resolution, with each grid cell spanning about 21.0 km^2^ [33]. To lessen spatial autocorrelation, we filtered the raw occurrence points with the spThin package in R; duplicates and records less than 5 km apart were discarded [34]. This yielded 1479 valid records of *P. castaneus*, largely distributed in Europe and southern South America (Figure 1).

#### 2.2.2. Selection and Screening of Environmental Variables

Because collinearity among environmental variables may weaken model prediction [35], we extracted climate values at all occurrence localities and ran Pearson correlation analysis in R 4.4.1. Then, together with MaxEnt jackknife tests, the percent contribution of each variable was computed. For any variable pair with |R| > 0.8, we kept the one with the larger percent contribution [36].

### 2.3. Optimization and Settings of the MaxEnt Model

Predictive accuracy of MaxEnt depends strongly on the regularization multiplier (RM) and feature combination (FC) [37]. FC classes usually comprise linear (L), quadratic (Q), hinge (H), product (P), and threshold (T). We tested eight FCs: L, LQ, LQH, LQHP, LQHPT, QHP, QHPT, and HPT, and eight RM settings: 0.5, 1.0, 1.5, 2.0, 2.5, 3.0, 3.5, and 4.0. Using the checkerboard2 approach in the ENMeval package (R 4.4.1), we computed the corrected Akaike information criterion (AICc) and chose the RM–FC pair with the lowest ΔAICc as optimal [38,39]. The *P. castaneus* occurrence records and the important environmental variables selected above were then run in MaxEnt 3.4.4. We randomly assigned 75% of records to training and 25% to testing. Ten runs with the same settings were performed, and their mean was used as the final output [36,37].

### 2.4. Division and Visualization of Suitable Areas

Outputs from the optimized MaxEnt model were loaded into ArcGIS 10.8 for reclassification and mapping. Suitable habitat for *P. castaneus* was divided by the natural breaks (Jenks) method into four levels: unsuitable (0–0.07), low (0.07–0.27), medium (0.27–0.53), and high (>0.53) [40,41]. The same software was applied to reclassify raster layers and compute the area of each level.

### 2.5. Model Accuracy Evaluation

Model performance was assessed with the area under the receiver operating characteristic curve (AUC) [40,42]. AUC ranges from 0 to 1, and larger values denote stronger prediction. In detail, AUC ≤ 0.6: no predictive ability; 0.6 < AUC ≤ 0.7: poor; 0.7 < AUC ≤ 0.8: fair; 0.8 < AUC ≤ 0.9: good; AUC > 0.9: very good discrimination [43,44].

### 2.6. Niche Dynamics Analysis

We examined niche dynamics for *P. castaneus* with the ecospat package under the COUE framework [45,46]. For current climate, the background calibration area was set from actual occurrence points plus a 1° buffer. First, known occurrences and the climate variables with major effects on *P. castaneus* distribution (Table 1) were retrieved from the Wallace platform in R for both current and future scenarios. A 1° buffer was created around each occurrence using Wallace, and 10,000 random background points were sampled inside these buffers [47,48]. Climate values were then extracted for occurrence and background points. On this basis, principal component analysis (PCA) was used to build a common environmental space (PCA-env) in multidimensional climate space, and the first two principal components were retained. Using occurrence data and environmental variables under each climate scenario, ecospat was applied to quantify niche dynamics and calculate future–current niche overlap. Overlap was measured by Schoener’s D, with larger values representing greater overlap [49]. We also ran niche similarity and equivalency tests with 1000 permutations; *p*-values indicated whether future and current niches were similar or equivalent [50]. Climatic niche dynamics were further separated into three parts: stability, expansion, and unfilling [29]. Specifically, expansion is the share of niche space present under future but absent under current climate; stability is the share of overlapping niche space between current and future; unfilling is the share of niche space present now but unoccupied in the future; overlap is the similarity/sharing between climatic niches occupied in native and introduced ranges [45,51]. Expansion or unfilling >0.1 was treated as substantial, allowing quantitative evaluation of niche change using these two indices [52,53].

## 3. Results

### 3.1. Parameter Settings of MaxEnt Model

The occurrence records of *P. castaneus* were subjected to SpThin package buffer analysis, and 1479 records from Europe, Asia, and South America were finally selected. Based on the screened distribution data, 10 key climatic factors were selected for suitable area prediction and ecological niche dynamics analysis (Table 2) through Pearson correlation analysis (Figure 2) and assessment of climatic factor contribution rates. The feature combination (FC) of the model was QHPT, and the regularization multiplier (RM) was 3 (Figure 3).

### 3.2. Evaluation of Model Prediction Accuracy

The predictive accuracy of the MaxEnt model was evaluated using the receiver operating characteristic (ROC) curve. Under current climate conditions, the mean AUC value from 10 replicates was 0.944 (Figure 4), and the ROC curve was steep, with sensitivity approaching 1 at a very low false positive rate. This indicates that the model can accurately distinguish suitable from unsuitable areas for the species. Under future climate scenarios, the AUC values of the MaxEnt model also exceeded 0.9 (Table 3), confirming that the model constructed in this study has high predictive accuracy and yields reliable results.

### 3.3. Prediction of Potential Geographical Distribution of P. castaneus Under Different Climate Conditions

#### 3.3.1. Prediction Under Current Climate Conditions

Under current climate conditions, the suitable areas for *P. castaneus* are mainly concentrated between 25° and 70° N and between 30° and 60° S (Figure 5). The total global potential suitable area of *P. castaneus* is approximately 1.08 × 10^7^ km^2^ (Figure 6), of which the low-suitability, medium-suitability, and high-suitability areas are 7.92 × 10^6^ km^2^, 1.39 × 10^6^ km^2^, and 1.46 × 10^6^ km^2^, respectively.

The prediction results (Figure 5) show that under current climate conditions, the high-suitability areas of *P. castaneus* are mainly distributed in Western Europe and northern Turkey, with smaller occurrences in Asia, southeastern Australia, the west coast of North America, and the southeastern coast of South America. Specifically, in Europe, high-suitability areas occur in most of the United Kingdom, Ireland, France, Italy, Germany, Greece, Andorra, Monaco, Denmark, Belgium, the Netherlands, Luxembourg, Albania, and Croatia, as well as parts of the Czech Republic, Austria, Portugal, Spain, Sweden, Poland, Bulgaria, and North Macedonia (Figure 5). In Asia, high-suitability areas are found along the Black Sea coast of northern Turkey and in the Kashmir region. In Oceania, they occur mainly in southeastern Australia (Figure 5). In North and South America, high-suitability areas are scattered in Argentina, Chile, Uruguay, Brazil, and parts of the west coasts of the United States and Canada (Figure 5). The moderately suitable areas are mainly distributed in Central Europe and southern Scandinavia, and are also scattered across parts of Asia, southeastern Australia, New Zealand, and the Americas, typically near the high-suitability areas (Figure 5). The low-suitability areas have the largest extent and are widely distributed along the east and west coasts of North America, across northern Eurasia, and in New Zealand, with smaller occurrences in East Asia, southern South America, and southeastern Australia (Figure 5).

#### 3.3.2. Prediction of Potential Global Geographical Distribution of *P. castaneus* Under Future Climate Conditions

Compared with current climate conditions, under the future SSP126 scenario, the total suitable area for *P. castaneus* shows a trend of first decreasing and then increasing. The total suitable areas for the four periods are 9.97 × 10^6^ km^2^, 1.06 × 10^7^ km^2^, 1.09 × 10^7^ km^2^, and 1.10 × 10^7^ km^2^, respectively (Figure 6a). Under this scenario, the maximum predicted area of the low-suitability area is 8.19 × 10^6^ km^2^, occurring in 2081–2100, an increase of 3.3% compared to current conditions. The minimum predicted low-suitability area is 7.23 × 10^6^ km^2^, occurring in 2021–2040, a decrease of 8.8% (Figure 6a). The moderately suitable area shows a similar trend of first increasing and then decreasing. Its maximum predicted area is 1.45 × 10^6^ km^2^, occurring in 2021–2040, an increase of 3.9% from current levels; the minimum is 9.79 × 10^5^ km^2^, occurring in 2061–2080, a decrease of 29.7% (Figure 6a). The highly suitable area exhibits a trend of first decreasing, then increasing, and then decreasing. Its maximum predicted area is 1.94 × 10^6^ km^2^, occurring in 2061–2080, an increase of 32.2% compared with the current climate conditions, while the minimum is 1.29 × 10^6^ km^2^, occurring in 2021–2040, a decrease of 11.9% (Figure 6a). Under the ssp126 scenario, changes in suitable areas for *P. castaneus* in Europe are particularly pronounced. Compared with current conditions, during 2021–2040, the high-suitability area in Western Europe slightly shrinks westward, with part of it converting to moderate suitability, while the low-suitability area remains largely unchanged (Figure 7a). During 2041–2060, the high-suitability area in Western Europe expands northeastward, with part of the moderate-suitability area converting to high suitability, while the low-suitability area remains stable (Figure 7b). Subsequently, during 2061–2080, the high-suitability area continues to expand eastward, converting part of the moderate-suitability area to high suitability and covering most of Europe (Figure 7c). However, during 2081–2100, the high-suitability area retreats westward (Figure 7d), and the distribution of high-suitability and moderate-suitability areas resembles that under current climate conditions.

Compared with current climate conditions, under the SSP585 scenario, the total suitable area for *P. castaneus* from 2021 to 2040 is 1.05 × 10^7^ km^2^ (Figure 6b), a decrease of 2.5%. During this period, the high-suitability area expands into Central Europe (e.g., Poland and the Czech Republic) and Northern Europe (e.g., Norway and Sweden) (Figure 8a), reaching 1.81 × 10^6^ km^2^, an increase of 23.1% from current levels. Under the SSP585 scenario, the total suitable area for *P. castaneus* from 2041 to 2060 remains similar to current conditions, covering 1.08 × 10^7^ km^2^ (Figure 6b and Figure 8b). During this period, the moderate-suitability area decreases to 9.85 × 10^5^ km^2^, which is 29.3% lower than the current climatic conditions, while the high-suitability area increases to 1.95 × 10^6^ km^2^, representing a 32.9% rise compared to the present-day baseline (Figure 6b). From 2061 to 2080, the total suitable area slightly declines to 1.067 × 10^7^ km^2^, a reduction of 1.0% from current levels; however, the high-suitability area reaches 1.77 × 10^6^ km^2^, an increase of 20.7% relative to current conditions (Figure 6b and Figure 8c). In the final period (2081–2100), the total suitable area attains its maximum of 1.13 × 10^7^ km^2^, which is 5.1% above the contemporary baseline (Figure 6b and Figure 8d). The moderate- and high-suitability areas are projected at 1.64 × 10^6^ km^2^ and 1.63 × 10^6^ km^2^, respectively, corresponding to increases of 17.7% and 11.4% from the current climatic conditions. At the same time, the low-suitability area also peaks at 8.07 × 10^6^ km^2^, a modest 1.7% increase relative to the present-day climate (Figure 6b).

#### 3.3.3. Relationship Between *P. castaneus* Distribution and Environmental Variables

In this study, the jackknife test was used to evaluate the relative importance of the key environmental variables selected for predicting the distribution of *P. castaneus*. The results show that among the 10 selected variables, the four with the greatest impact on the distribution of *P. castaneus* are mean temperature of the coldest quarter (bio11), precipitation of the driest month (bio14), precipitation seasonality (bio15), and the standard deviation of temperature seasonality (bio04) (Figure 9).

This study plotted the response curves of these four key environmental variables and analyzed the relationship between the survival probability of *P. castaneus* and changes in these climate variables (Figure 10). The most suitable range of bio11 for *P. castaneus* is 0.368–8.321 °C, with an optimal mean temperature of 5.82 °C. Within 0.368–5.82 °C, the existence probability of *P. castaneus* increases with temperature, but decreases from 5.82 to 8.321 °C (Figure 10a). The most suitable range of bio14 for *P. castaneus* is 34.564–79.608 mm. Its existence probability reaches the highest within the range of 42.541–58.493 mm. *P. castaneus* occurrence probability increases with precipitation over 34.564–42.541 mm and decreases over 58.493–79.608 mm (Figure 10b). The most suitable range of bio15 for *P. castaneus* is 8.628–26.065, and the predicted existence probability is highest at 12.169 (Figure 10c). The most suitable range of the bio04 for *P. castaneus* is 408.968–658.841. Within this range, the existence probability of *P. castaneus* first increases and then decreases with increasing bio04: from 408.968 to 535.309, it increases; from 535.309 to 658.841, it decreases (Figure 10d).

### 3.4. Niche Overlap Analysis

Niche dynamics analysis using the COUE frame showed that the niche overlap values for *P. castaneus* range from 0.467 to 0.841 (Table 4). Under SSP126, niche stability value ranged from 0.976–0.988, while niche expansion and unfilling remained low, ranging from 0.012 to 0.024 and from 0.011 to 0.027, respectively. Under SSP585, Schoener’s D value decreased steadily from 0.766 in 2021–2040 to 0.467 in 2081–2100. Niche stability values also declined, from 0.979 to 0.771, falling below 0.9 in both 2061–2080 and 2081–2100. Meanwhile, niche expansion and unfilling values increased from 0.021 to 0.229 and from 0.019 to 0.093, respectively (Table 4). These results indicate that the greater changes in the climatic niche of *P. castaneus* under SSP585, particularly toward the end of the century.

Niche equivalency tests reveal that the future niches of *P. castaneus* are not equivalent to its current niche under either the SSP126 (*p* < 0.05) or SSP585 scenario (*p* < 0.05, Figure 11), whereas niche similarity tests reject the null hypothesis (*p* < 0.05), indicating that the species’ niche retains some degree of similarity under climate change.

## 4. Discussion

### 4.1. Species Distribution Model

Previous studies have demonstrated that using the default parameters of MaxEnt frequently yields overly complex models that are prone to overfitting the existing distribution data, thereby introducing biases into predictions of species’ potential suitable areas [54,55,56,57]. To mitigate this issue, the present study employed the model parameter tuning function of the ENMeval package to systematically optimize the regularization multiplier and feature combination, thereby reducing model complexity, suppressing overfitting [37,56,57,58]. Under current climate conditions, the predictions generated by the MaxEnt model built with the optimized parameter set align well with the actual European distribution of *P. castaneus* (Figure 5), and the AUC value is greater than 0.9 (Table 3), indicating that the optimization not only reduces model complexity but also substantially improves prediction accuracy.

### 4.2. Evolutionary Pattern of the Distribution Area of P. castaneus Under Current and Future Climate Conditions

The prediction results of this study show that under current climate conditions, the high-suitability areas for *P. castaneus* are mainly located in Western Europe and northern Turkey (Figure 5), indicating that the high-suitability regions largely cover the temperate oceanic climate zones. Temperate oceanic climates are characterized by a coldest-quarter mean temperature > 0 °C, moist throughout the year, even rainfall distribution, and small monthly temperature fluctuations [59,60]. This study, through Jackknife analysis, revealed that the four environmental variables most influential on the distribution of the *P. castaneus* were bio11, bio14, bio15 and bio04 (Figure 9). The most suitable range of bio11 for the survival of *P. castaneus* is 0.368–8.321 °C, which is greater than 0 °C; The most suitable range of bio14 for *P. castaneus* is 34.564–79.608 mm, which also meets the criteria for a humid climate year-round.; For the survival of *P. castaneus*, the most suitable ranges of bio15 and bio04 are 8.628–26.065 and 408.968–658.841, respectively. These values are also consistent with the characteristics of a temperate maritime climate. At present, relatively few studies have examined the effects of environmental factors on the biology of *P. castaneus*. However, studies on its congener *P. strobi* have shown that temperature can affect larval feeding, mating, and oviposition behavior [61]. Meanwhile, extreme water stress can negatively affect the hosts of *P. strobi*, potentially leading to its outbreak [62]. Therefore, we speculate that extreme low temperatures may reduce the overwintering survival rates of the autumn-generation larvae, as well as the spring-generation and previous-year adults of *P. castaneus*, thereby affecting the production of spring-generation eggs in the following year. However, bio05 provides the most unique information for the distribution of *P. castaneus* (Figure 9). Extreme high temperatures may affect the mating and oviposition behavior of the spring- and autumn-generation adults of *P. castaneus* by reducing the frequency of mating activity. In addition, extreme precipitation may indirectly affect the suitable habitat of *P. castaneus* by imposing stress on its coniferous tree hosts.

The Sixth Assessment Report of the Intergovernmental Panel on Climate Change (IPCC AR6) indicates that by the end of the 21st century, the global average temperature increase may exceed the 1.5 °C threshold [31]. The future climate scenario data used in this study are derived from the BCC-CSM2-MR global climate model under the Coupled Model Intercomparison Project Phase 6 (CMIP6) [31]. SSP126 and SSP585 were selected for suitability area simulation analysis. SSP126 is an updated version of the RCP2.6 scenario, with radiative forcing stabilizing at 2.6 W/m^2^ by 2100, representing a low-emission, low-forcing scenario [31,63]; SSP585 corresponds to an upgraded version of RCP8.5, with radiative forcing maintained at 8.5 W/m^2^ by 2100, representing a high-emission, high-forcing extreme climate scenario [64]. Relative to the current climate conditions, under the two different future climate scenarios (SSP126 and SSP585) with distinct carbon emission pathways, the total suitable area for *P. castaneus* generally exhibits a trend of first decreasing and then increasing (Figure 6); Moreover, the level of carbon emissions also influences the spatial distribution of suitable areas. Notably, under both future scenarios, the northern boundary of the suitable range of *P. castaneus* shows a northward migration trend. Similar phenomena have been observed in studies on other insects [65,66], such as *Phenacoccus solenopsis* and *Loxostege sticticalis,* indicating that climate-driven northward migration of suitable insect habitats is a relatively common occurrence.

The prediction results of the potential distribution of *P. castaneus* under the current and future climate conditions indicate that future suitable areas for this species will migrate significantly northeastward in Europe compared to the present situation. Consequently, Central European countries such as Poland and the Czech Republic, as well as Nordic countries including Norway and Sweden, should strengthen monitoring and early warning systems for this pest and implement necessary control measures. Meanwhile, most of Western Europe will remain a highly suitable area for *P. castaneus* in the future, requiring further reinforcement of quarantine efforts to prevent population outbreaks and large-scale spread. Additionally, potential suitable areas for *P. castaneus* also exist in North America, Australia, and New Zealand under both current and future climate conditions. Although no actual invasion of *P. castaneus* has yet been recorded in these areas, given its potential ecological and economic threats, future quarantine inspections of imported wood products should focus on detecting this pest to prevent its introduction and establishment. In conclusion, based on the projected potential distribution of *P. castaneus* under current and future climate scenarios, differentiated quarantine policies are recommended for distinct geographical regions. For Central European countries (e.g., Poland and the Czech Republic) and Nordic countries (e.g., Norway and Sweden), where suitable areas are projected to shift significantly northeastward, priority should be given to strengthening entry quarantine inspections and early monitoring and early warning systems, particularly by intensifying scrutiny of timber and wood packaging materials originating from Western Europe—a region that remains persistently highly suitable—and by proactively listing this pest as a potentially dangerous quarantine organism. For Western Europe, which consistently maintains high suitability, sustained efforts should focus on reinforcing export quarantine and in-region population surveillance to prevent outbreaks and onward spread, while actively participating in the harmonization of regional quarantine protocols. For North America, Australia, and New Zealand, where no introduction has been recorded but climatic conditions are potentially suitable, a preventive quarantine strategy should be adopted, designating *P. castaneus* as a key regulated pest for imported goods, strictly enforcing approved phytosanitary treatments on consignments from Europe, and establishing rapid response protocols for interceptions to prevent its entry and subsequent establishment.

### 4.3. Niche Overlap Analysis of P. castaneus

To some extent, a species’ distribution range is determined by its fundamental niche—the set of environmental conditions under which it can survive and reproduce [67]. However, owing to geographic or biological barriers, a species may not occupy all areas with suitable abiotic conditions, but rather only a subset of its fundamental niche hypervolume, referred to as its ‘realized niche’ [67]. This study used the COUE framework to compare the ecological niches of *P. castaneus* under current and future climate scenarios to clarify the dynamic characteristics of its ecological niche under climate change. The results show that, under the SSP126 scenarios, *P. castaneus* maintains high niche stability (>0.9, Table 4) and a low proportion of unfilled niches (<0.1, Table 4), retaining the ecological niche characteristics under the current climate conditions. However, under the SSP585 scenario, Schoener’s D for *P. castaneus* decreases. During 2081–2100, niche stability was lowest, while niche expansion and unfilling values were highest (Table 4). It suggested that the species may utilize novel environmental conditions under an extreme high-emission scenario. Li et al. found that niche shifts in *Agrilus planipennis* were more pronounced under a high-carbon emission scenario than under a low-carbon emission scenario [68], showing patterns similar to those observed in the present study. Different carbon emission scenarios may exert differential impacts on the ecological niche of *P. castaneus*, but the relatively high niche stability (>0.7) and similarity test results indicate that the niche of *P. castaneus* will still exhibit a certain degree of conservatism under future climate scenarios.

## 5. Conclusions

This study predicts the global potential geographic distribution of *P. castaneus* for the first time under current and future climate change scenarios. Under current climate conditions, highly suitable areas of *P. castaneus* are mainly concentrated in Western Europe, northern Turkey, southeastern Australia, the west coast of North America, and the east coast of South America. Under future climate scenarios, the total potential distribution area is projected to initially decrease and then increase, with a notable northward shift in Europe toward higher latitudes. We also identified several key climatic factors affecting the potential distribution of *P. castaneus*. More importantly, niche dynamics analysis revealed that although *P. castaneus* exhibits some degree of niche conservatism, the increased expansion and unfilled niche values under future extreme high-emission scenarios suggest that this pest may exploit novel environmental conditions and thus have potential for global spread and establishment. This research provides certain scientific basis for the management of *P. castaneus* and the formulation of comprehensive control measures.

## Figures and Tables

**Figure 1:**
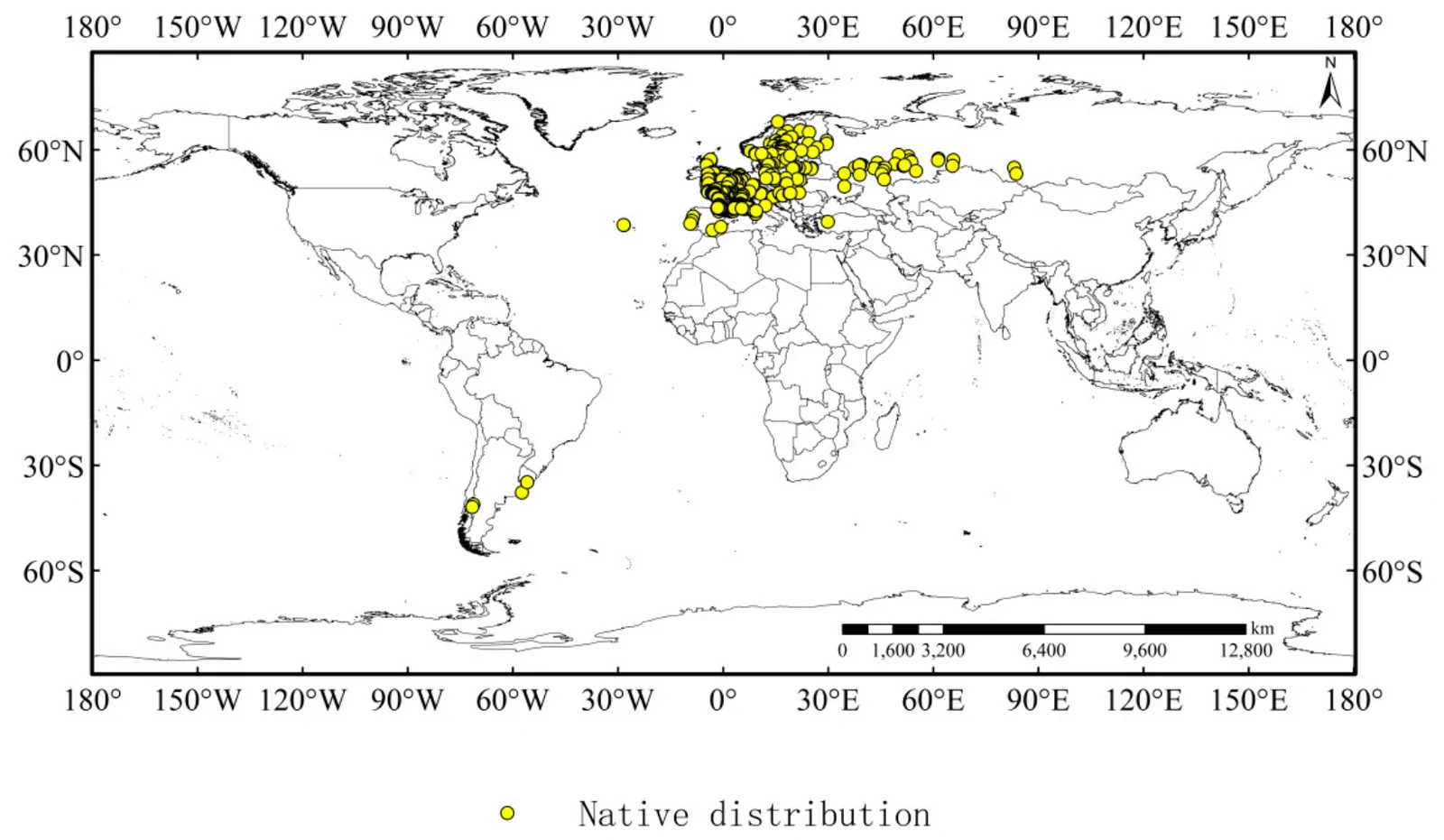
Occurrence records and global distribution of *P. castaneus*.

**Figure 2:**
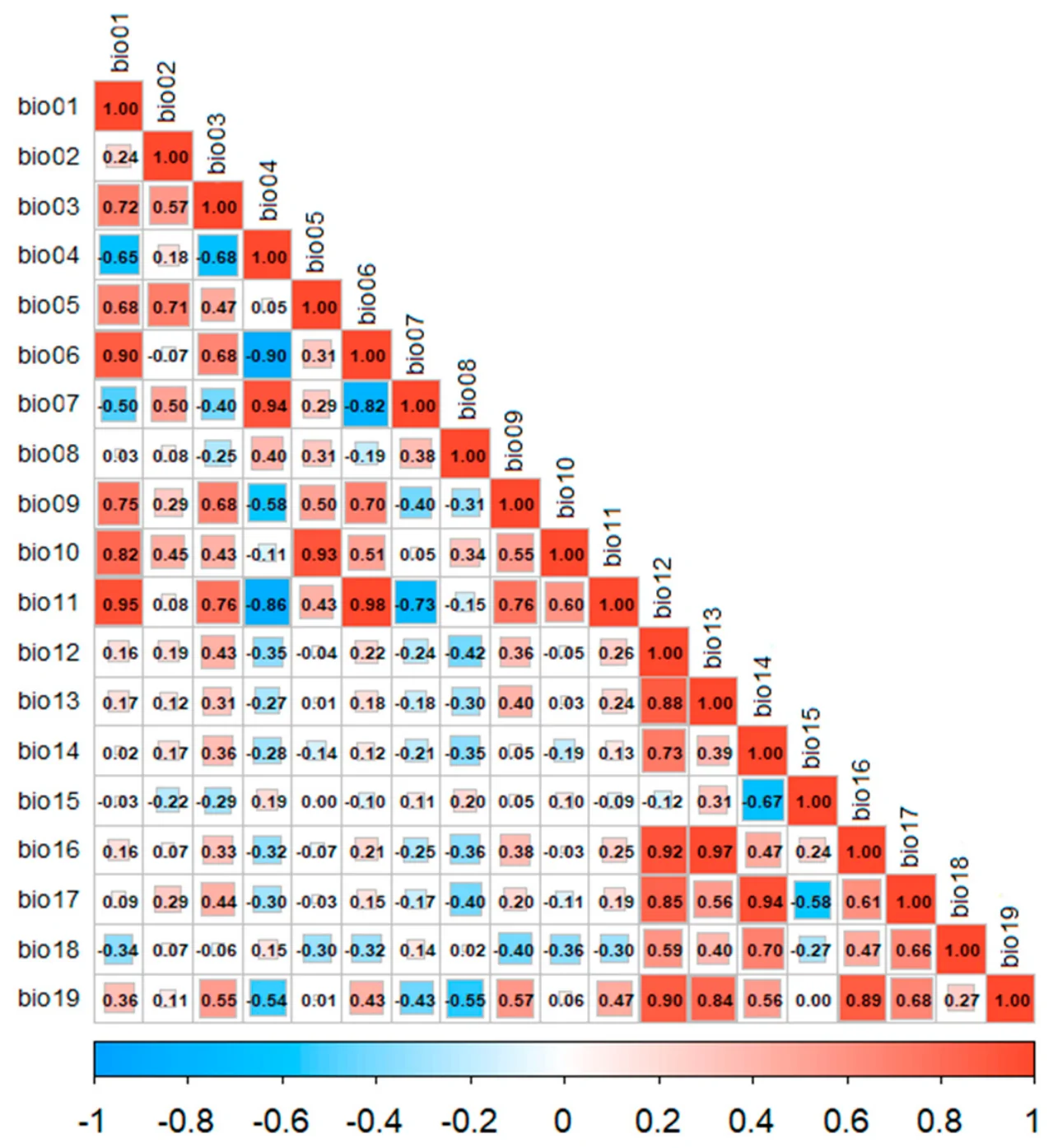
Correlations among the bioclimatic variables. The size and color depth of the squares indicate the magnitude of the correlation coefficient. The larger the area and the darker the color of the blue square, the stronger the negative correlation between the climatic variables; The larger the area and the darker the color of the red square, the stronger the positive correlation between the climatic variables; climate factors with a correlation coefficient |*R*| > 0.8 were retained.

**Figure 3:**
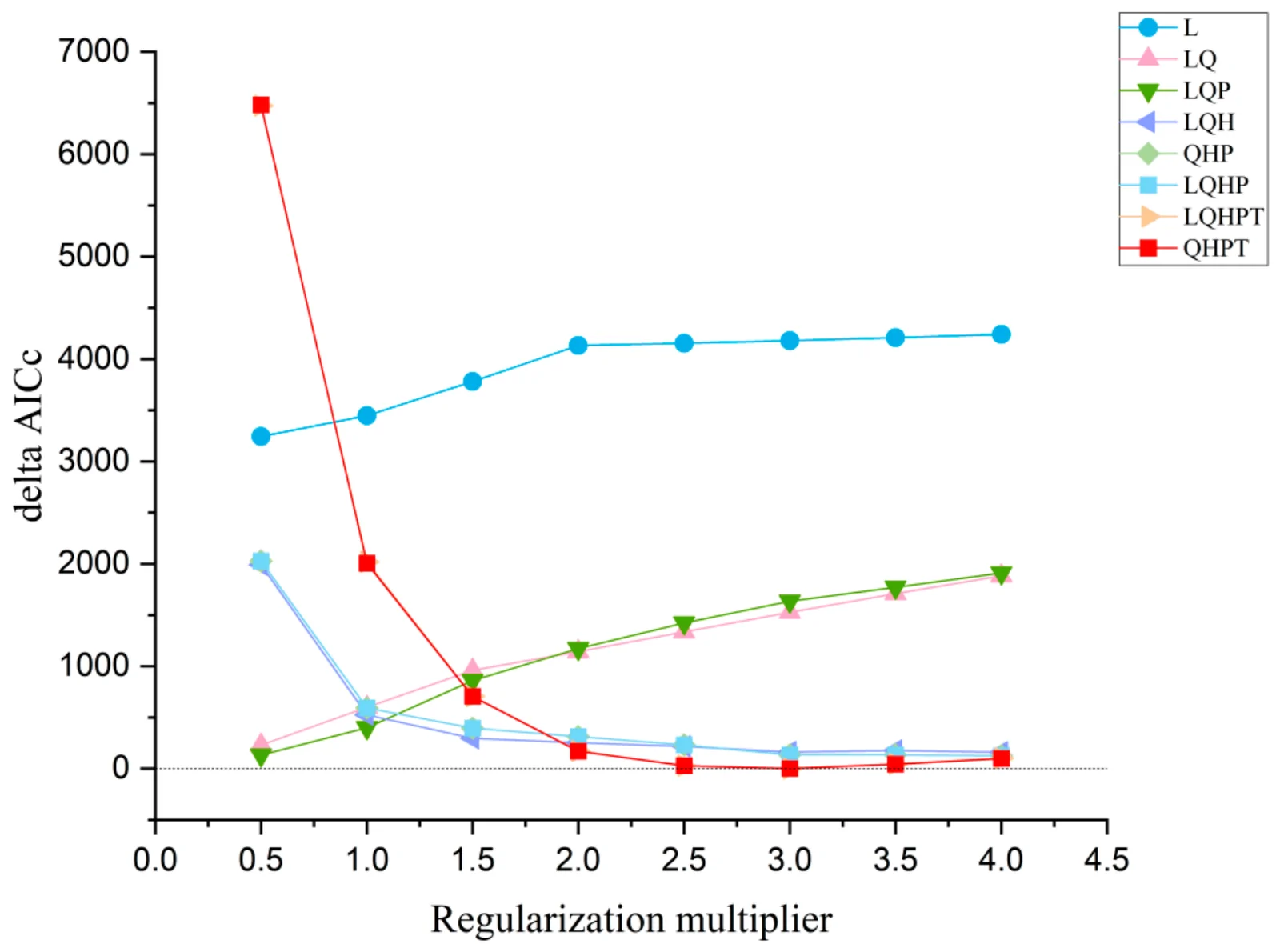
The regularization multiplier and feature classes of *P. castaneus* in the MaxEnt model. The dotted line represents the situation where the delta AICc value is 0.

**Figure 4:**
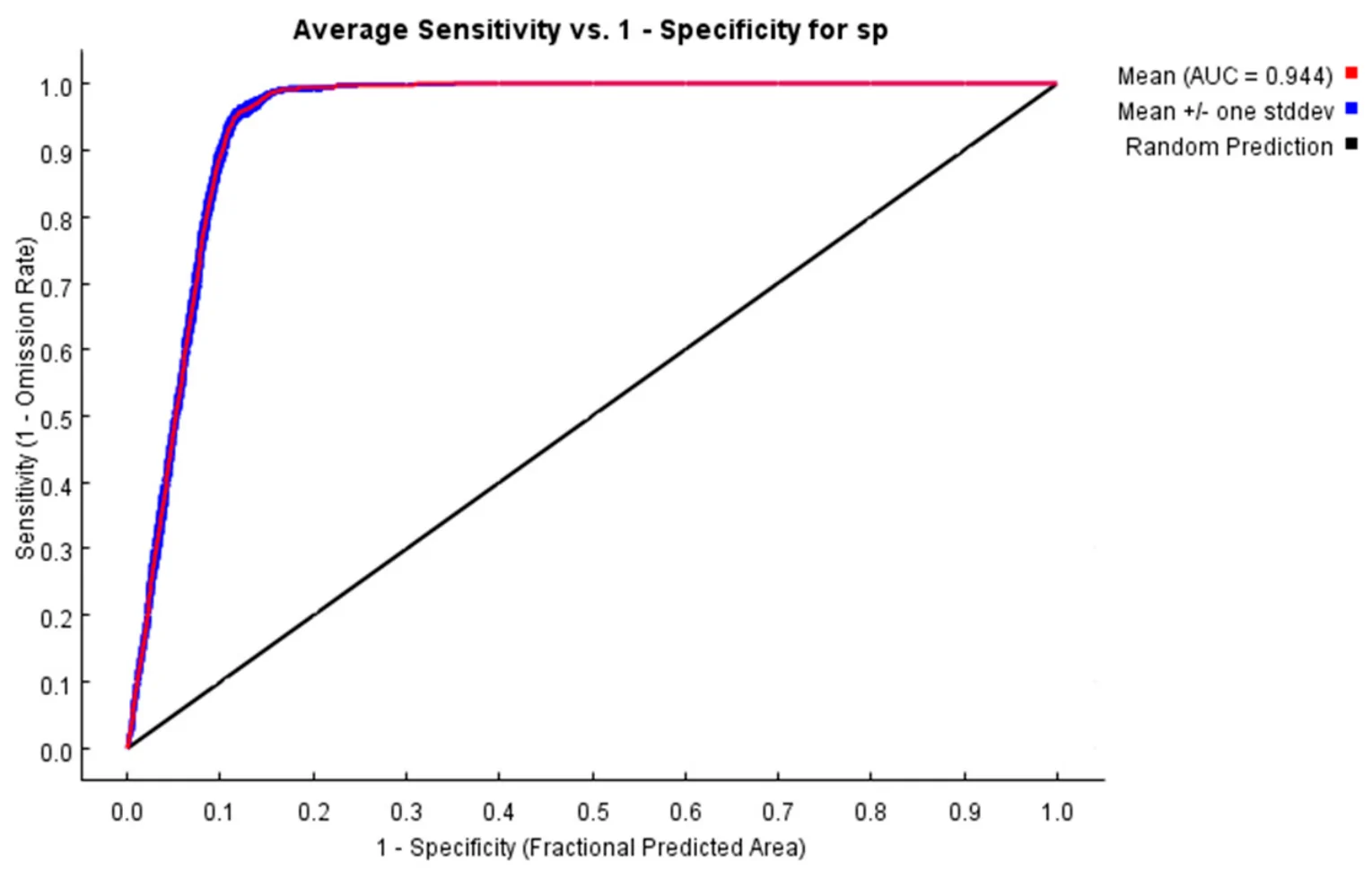
ROC curve for the potential suitable areas of *P. castaneus*. The ROC curve plots sensitivity (true positive rate) on the vertical axis against 1 − specificity (false positive rate) on the horizontal axis; the area under the curve (AUC) serves as the evaluation metric for the model’s predictive accuracy.

**Figure 5:**
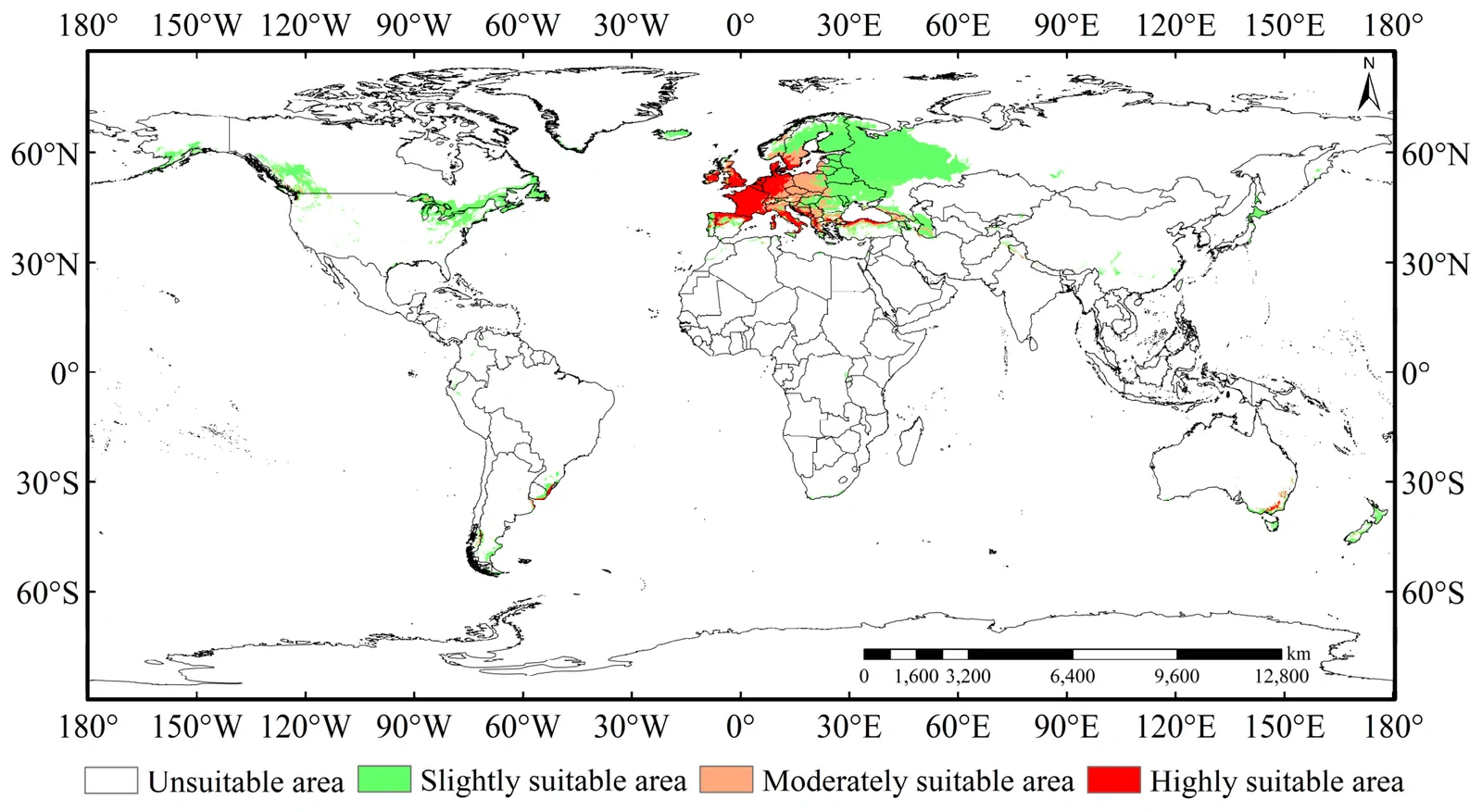
Potential global geographical distribution of *P. castaneus* under current climate conditions. On the map, green indicates low-suitability areas, orange indicates moderate-suitability areas, and red indicates high-suitability areas.

**Figure 6:**
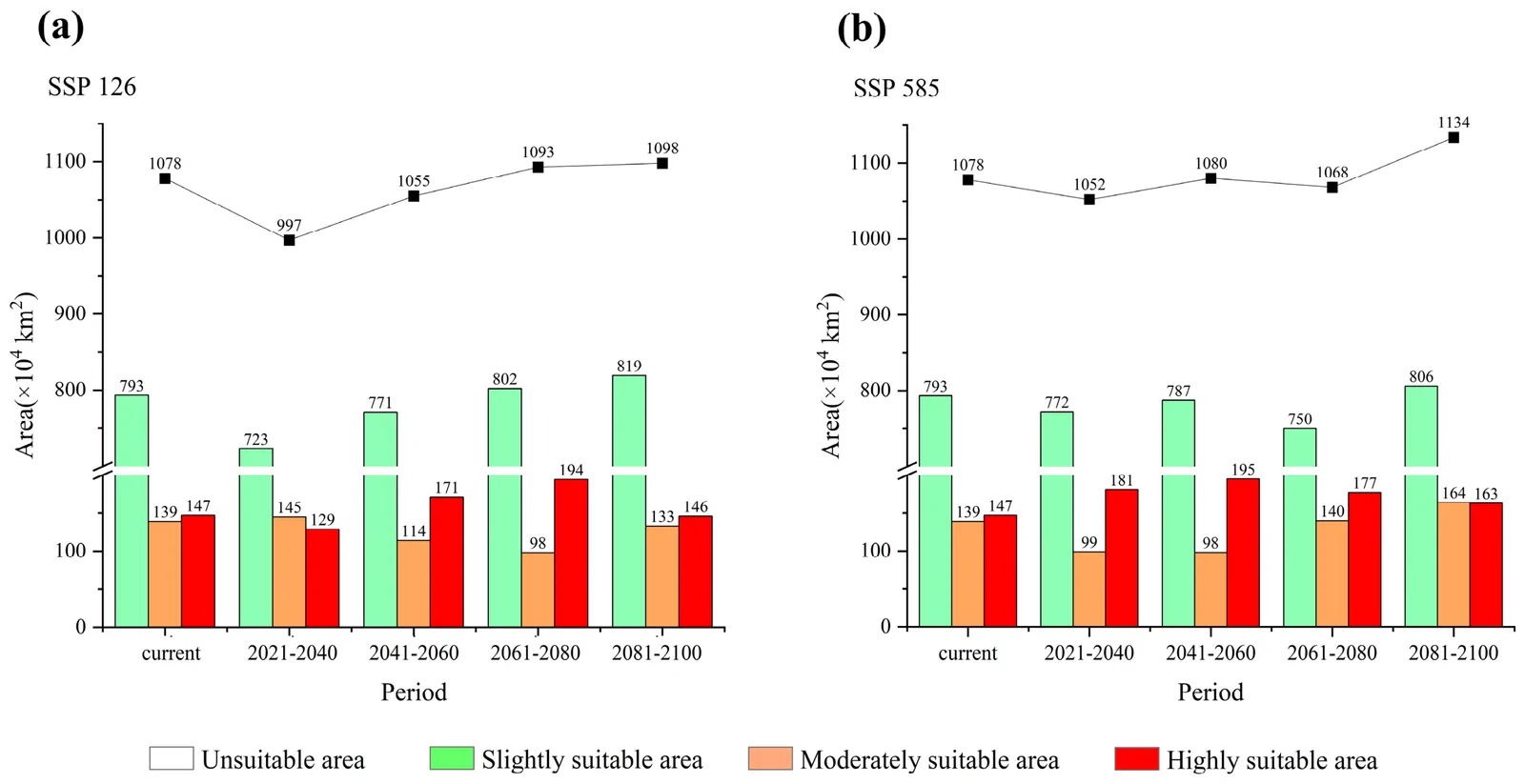
Areal changes (×10^4^ km^2^) of unsuitable, moderately suitable, and highly suitable habitats for *P. castaneus* under SSP126 and SSP585 scenarios from 2021 to 2100. Comparison between the predicted coverage area of the potential *P. castaneus* under current and future climate conditions for scenarios (**a**) SSP126 and (**b**) SSP585.

**Figure 7:**
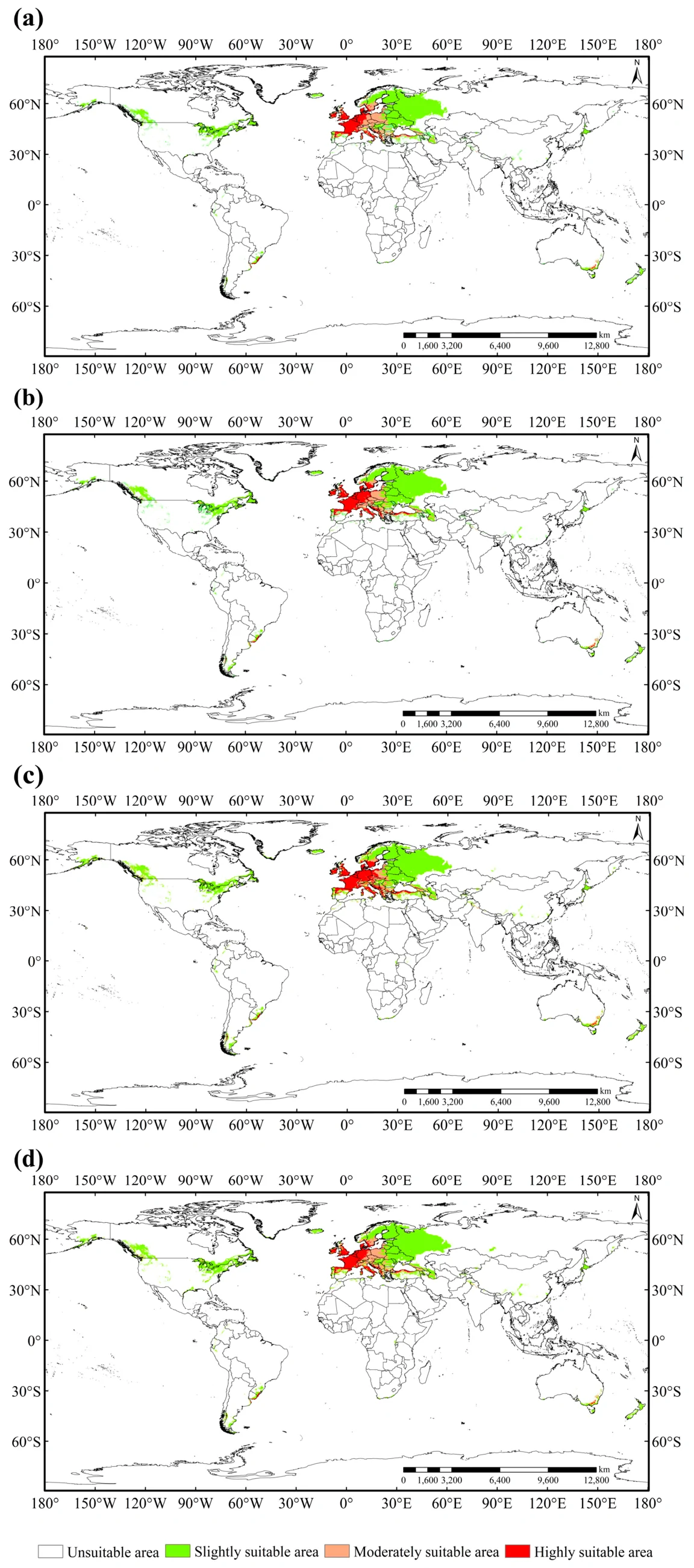
Predicted global suitable areas for *P. castaneus* under SSP126 for four periods: (**a**) 2021–2040, (**b**) 2041–2060, (**c**) 2061–2080, and (**d**) 2081–2100. Green, orange, and red indicate low, moderate, and high suitability, respectively.

**Figure 8:**
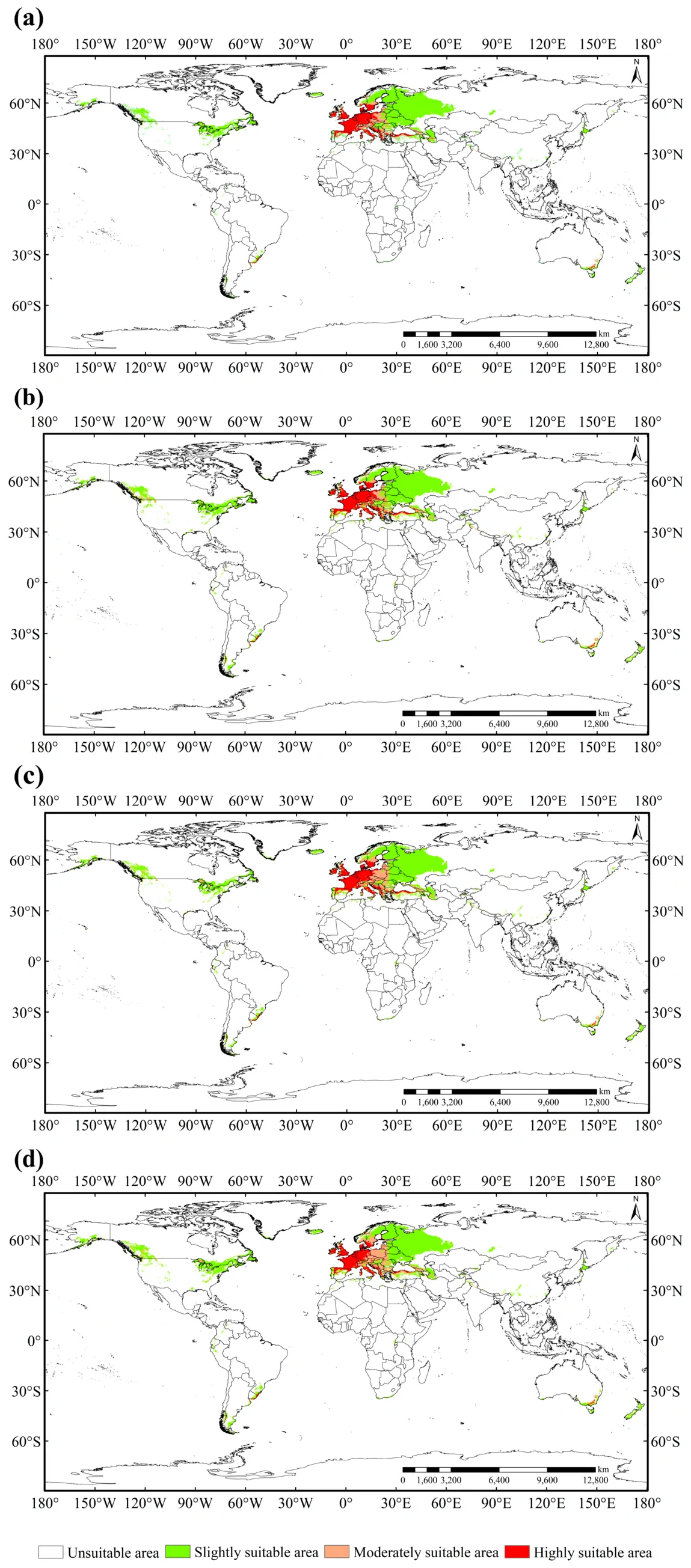
Predicted global suitable areas for *P. castaneus* under SSP585 for four periods: (**a**) 2021–2040, (**b**) 2041–2060, (**c**) 2061–2080, and (**d**) 2081–2100. Green, orange, and red indicate low, moderate, and high suitability, respectively.

**Figure 9:**
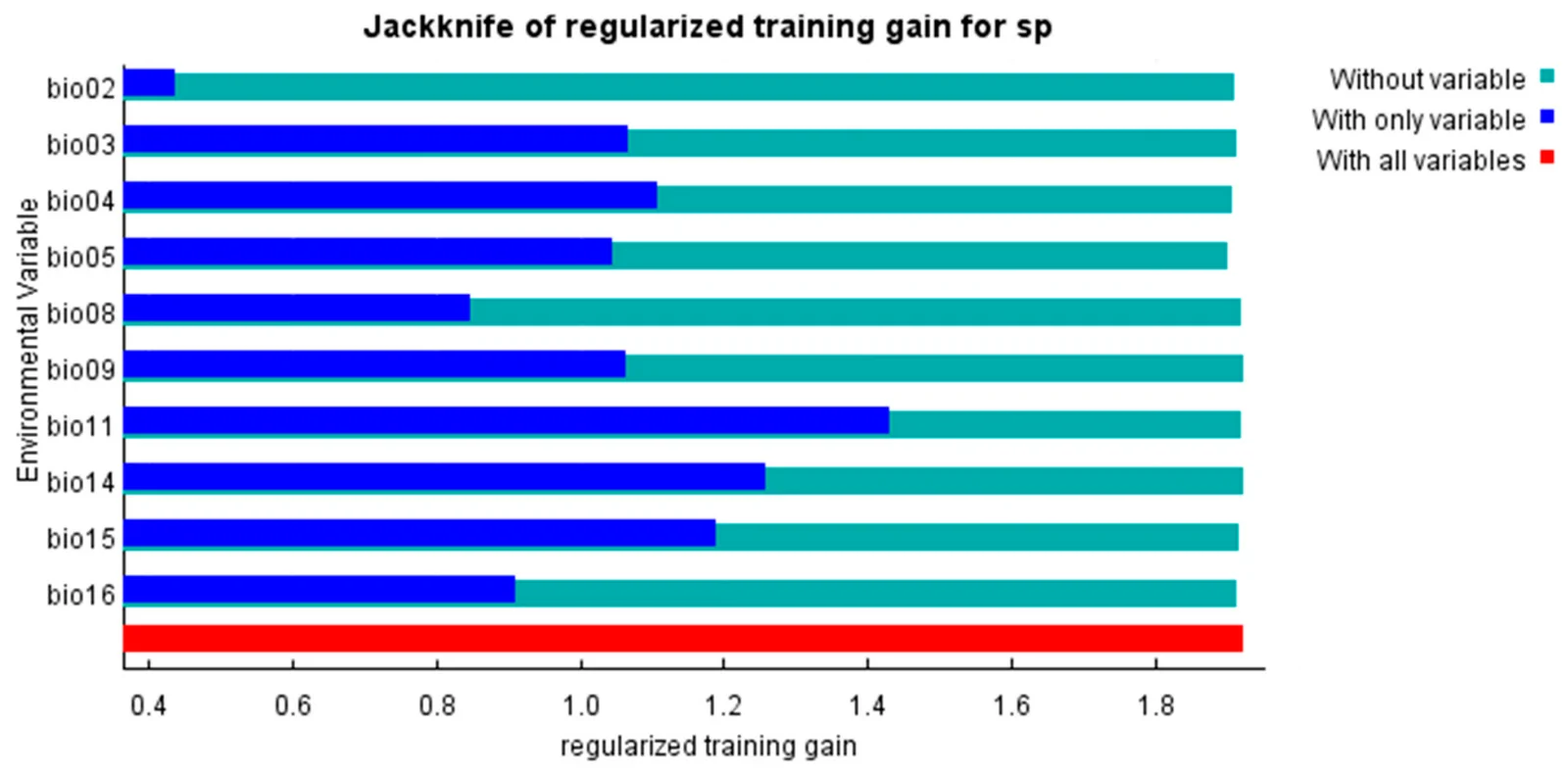
Jackknife analysis result showing most important environmental variables predicting potentially suitable distribution areas of *P. castaneus.* Blue bars represent the training gain when using only a single variable to build the model. The longer the bar, the stronger the variable’s ability to explain the species distribution alone. Green bars represent the training gain when the variable is excluded and the model is built using the remaining variables. The shorter the bar, the more unique information the excluded variable contains—information that cannot be replaced by other variables.

**Figure 10:**
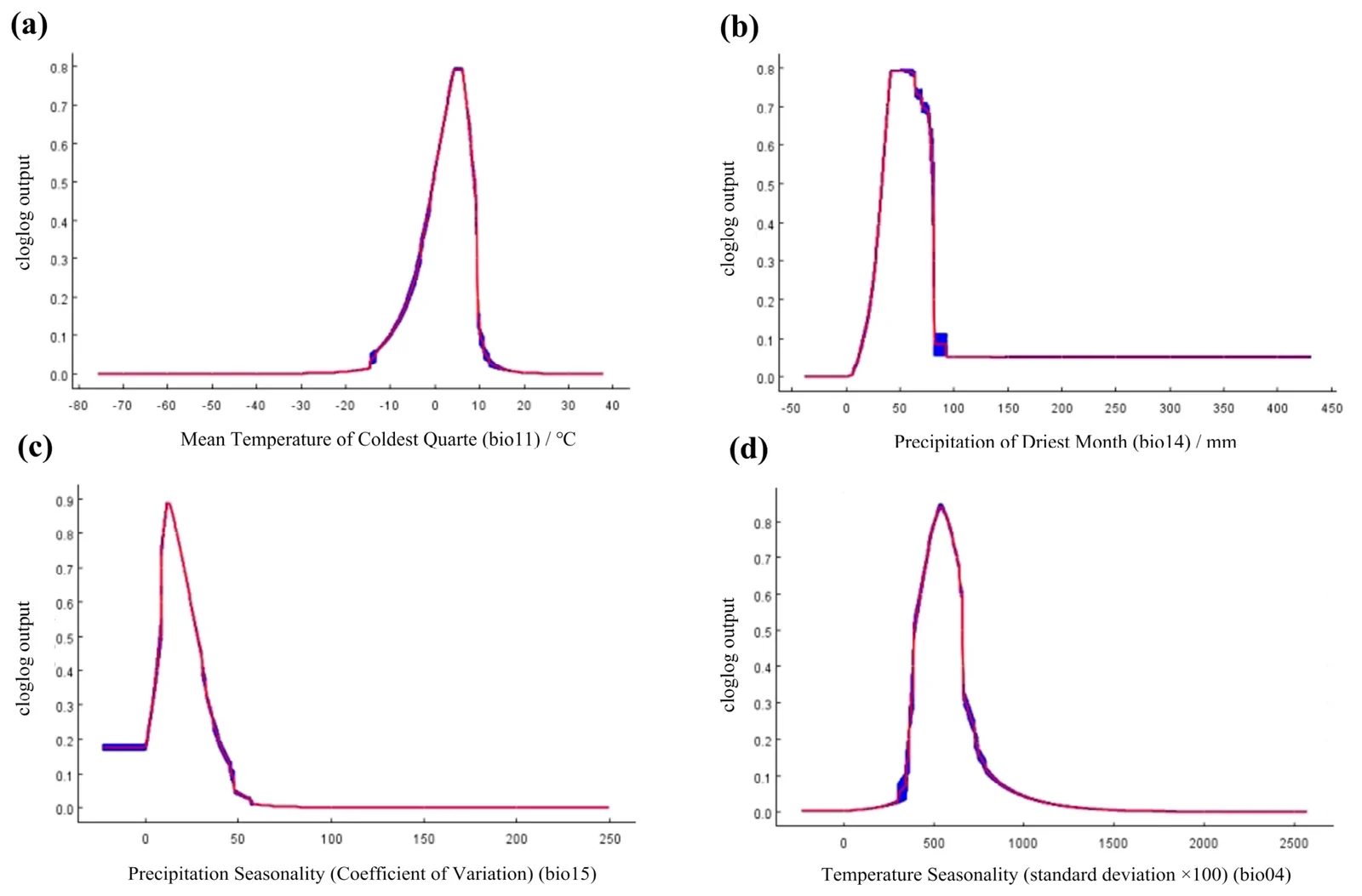
Response curves between the distribution probability of *P. castaneus* and four environmental variables: (**a**) mean temperature of the coldest quarter (bio11), (**b**) precipitation of the driest month (bio14), (**c**) precipitation coefficient of variation (bio15), and (**d**) the standard deviation of temperature seasonality (bio04).

**Figure 11:**
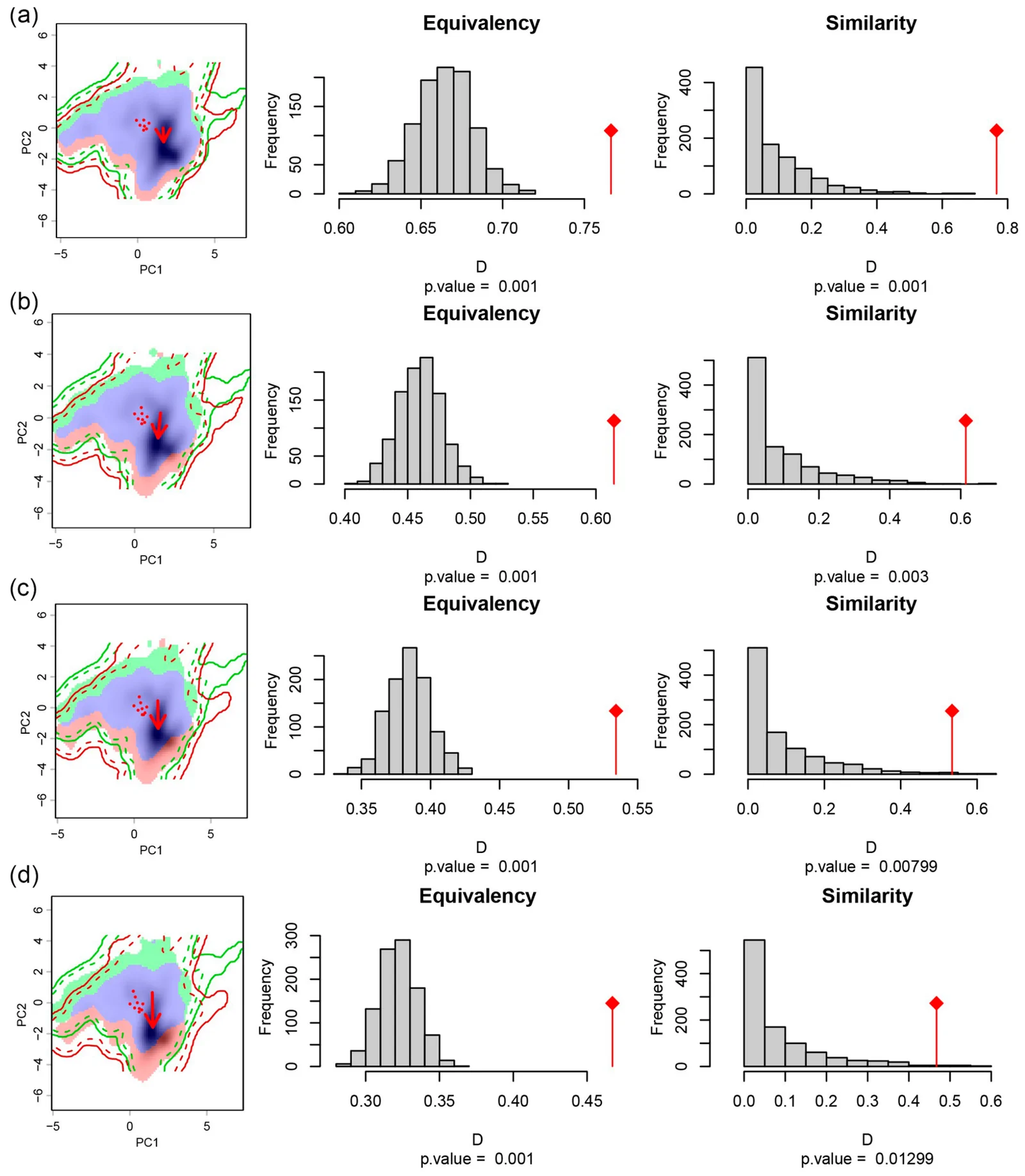
Analysis diagram of ecological niche changes in *P. castaneus* under the SSP585 scenario. Figures, respectively, depict the ecological niche shifts in *P. castaneus* under the SSP585 scenario for the periods: (**a**) 2021–2040, (**b**) 2041–2060, (**c**) 2061–2080, and (**d**) 2081–2100. The left half of each subfigure illustrates the temporal dynamics of the species’ ecological niche across the respective periods. Green and red shadings denote the occurrence density of *P. castaneus* under current and future climate scenarios, while purple shading indicates the overlapping regions of niche space between the two scenarios. Red arrows represent the directional shift in the climatic niche from current to future climate conditions. The right half presents the results of niche equivalency tests and niche similarity tests, which quantify the degree of niche overlap and divergence between the current and future scenarios. Among them, the red markers in the figure represent the actual Schoener’s D values that have been observed.

**Table 1:** The 10 key climatic factors used for the construction of the MaxEnt model.

Code	Full Name and Ecological Meaning
bio02	Mean Diurnal Range (Mean of monthly (max temp–min temp))
bio03	Isothermality (bio2/bio7) (×100)
bio04	Temperature Seasonality (standard deviation ×100)
bio05	Max Temperature of Warmest Month
bio08	Mean Temperature of Wettest Quarter
bio09	Mean Temperature of Driest Quarter
bio11	Mean Temperature of Coldest Quarter
bio14	Precipitation of Driest Month
bio15	Precipitation Seasonality (Coefficient of Variation)
bio16	Precipitation of Wettest Quarter

**Table 2:** Environmental variables used to predict the potential geographic distribution of *P. castaneus*.

Climatic Factors	Percent Contribution
bio15	27.2827
bio14	16.1404
bio04	14.0834
bio11	13.5982
bio05	9.0925
bio03	7.8486
bio08	5.5496
bio02	3.7645
bio16	2.5923
bio09	0.0479

**Table 3:** AUC values of the MaxEnt model under various climate conditions.

Climate Scenarios	AUC
Near current	0.944
2021–2040 ssp126	0.943
2041–2060 ssp126	0.942
2061–2080 ssp126	0.944
2081–2100 ssp126	0.945
2021–2040 ssp585	0.944
2041–2060 ssp585	0.942
2061–2080 ssp585	0.945
2081–2100 ssp585	0.944

**Table 4:** Ecological niche indicators of *P. castaneus* under different climate models.

	Niche Indicators	D.overlap	Expansion	Stability	Unfilling
Time Period					
SSP126 2021–2040		0.841	0.012	0.988	0.011
SSP126 2041–2060		0.773	0.014	0.986	0.021
SSP126 2061–2080		0.789	0.016	0.984	0.017
SSP126 2081–2100		0.738	0.024	0.976	0.027
SSP585 2021–2040		0.766	0.021	0.979	0.019
SSP585 2041–2060		0.614	0.066	0.934	0.049
SSP585 2061–2080		0.534	0.145	0.855	0.070
SSP585 2081–2100		0.467	0.229	0.771	0.093

## Data Availability

The data that support the findings of this study are available from the corresponding authors upon reasonable request.

## Supplementary Materials

The following supporting information can be downloaded at https://www.mdpi.com/article/10.3390/insects17101002/s1, Table S1: Full Name and Ecological Meanings of 19 bioclimatic Factors Code.
