# Supplementary material for: Potential Geographical Distribution Prediction and Ecological Niche Change Analysis of Pissodes castaneus
Source: Insects. 2026 Sep 28;17(10):1002. doi: 10.3390/insects17101002 (PMC13705476; doi:10.3390/insects17101002)
Supplement: Supplementary file 1 [file insects-17-01002-s001.zip › insects-4531355-supplementary.pdf]

Table S1. Full Name & Ecological Meanings of 19 bioclimatic Factors Code.

Code	Full Name & Ecological Meaning
bio01	Annual Mean Temperature
bio02	Mean Diurnal Range (Mean of monthly (max temp - min temp))
bio03	Isothermality (bio2/bio7) (×100)
bio04	Temperature Seasonality (standard deviation ×100)
bio05	Max Temperature of Warmest Month
bio06	Min Temperature of Coldest Month
bio07	Temperature Annual Range (bio5-bio6)
bio08	Mean Temperature of Wettest Quarter
bio09	Mean Temperature of Driest Quarter
bio10	Mean Temperature of Warmest Quarter
bio11	Mean Temperature of Coldest Quarter
bio12	Annual Precipitation
bio13	Precipitation of Wettest Month
bio14	Precipitation of Driest Month
bio15	Precipitation Seasonality (Coefficient of Variation)
bio16	Precipitation of Wettest Quarter
bio17	Precipitation of Driest Quarter
bio18	Precipitation of Warmest Quarter
bio19	Precipitation of Coldest Quarter
